# Miller Fisher Variant of Guillain-Barré Syndrome: A Great Masquerader

**DOI:** 10.7759/cureus.11045

**Published:** 2020-10-19

**Authors:** Kinnera Sahithi Urlapu, Muhammad Saad, Peter Bhandari, Jared Micho, Muhammad T Hassan

**Affiliations:** 1 Internal Medicine, BronxCare Health System, Bronx, USA; 2 General Internal Medicine, BronxCare Health System, Bronx, USA; 3 Internal Medicine, American University of the Caribbean School of Medicine, Sint Maarten, SXM; 4 Biology, Stony Brook University, New York, USA

**Keywords:** miller fisher syndrome, guillain-barré, ascending paralysis, anti-gq1b antibodies, demyelinating neurological disorder

## Abstract

Guillain-Barré Syndrome (GBS) is an acquired degenerative, demyelinating neurological disorder classically characterized by progressive, symmetrical ascending paralysis. Often associated to occur after a viral illness, most commonly an upper respiratory infection (URI), followed by gastrointestinal illnesses. Here we present a case of Miller Fisher syndrome (MFS) which is a rare variant of GBS. MFS presents with a triad of ataxia, areflexia, and opthalmoplegia. MFS is a clinical diagnosis but can be confirmed serologically with positive anti-ganglioside antibodies.

## Introduction

Guillain-Barré Syndrome (GBS) is an acquired degenerative, demyelinating neurological disorder classically characterized by progressive, symmetrical ascending paralysis. Absent muscle reflexes and loss of sensation are also commonly associated [1]. The etiology remains unclear, but onset has been associated with viral illness, most commonly an upper respiratory infection (URI), followed by gastrointestinal illness [2]. *Campylobacter jejuni* and *Haemophilus influenza* are the most commonly involved pathogens [3]. 

Miller Fisher Syndrome (MFS) is a rare variant of GBS, observed in only about 1-5% of all cases of GBS in Western countries [2-4]. In other geographic regions such as Taiwan and Japan, the incidence is up to 19% and 25%, respectively [2]. MFS presents with a clinical triad of ataxia, areflexia, and ophthalmoplegia [2,5]. One of the main differences between MFS and the other, more common variants of GBS is that the first nerve groups to demyelinate are commonly located in the cranium. This results in difficulties with balance and coordination, ocular muscle movement and vision impairment, and neuronal reflexes [3]. MFS is a clinical diagnosis but often goes undiagnosed due to the low prevalence. MFS is a clinical diagnosis that can be confirmed serologically with positive anti-ganglioside GQ1b antibodies. Here, we present a rare case of MFS and the importance of having a high index of suspicion in an acutely symptomatic patient.

## Case presentation

Our patient is a 44-year-old Hispanic woman who presented to the emergency room (ER) complaining of unilateral right-sided ptosis for two days. She also reported having diplopia and blurry vision initially. She did not have any headaches, seizures, eye discharge, changes in speech, weakness in her extremities, trauma or recent viral illness. She also has a medical history significant for type 2 diabetes mellitus, hypertension, obstructive sleep apnea and obesity. No significant surgical or family history. Patient denied any toxic habits including smoking, alcohol or illicit drug use. Her initial vital signs in the ER demonstrated an afebrile patient, with a blood pressure of 130/85 mmHg, pulse of 66 beats/min, respiratory rate of 17 breaths/min and an O2 saturation of 99% on room air. Her BMI (Body Mass Index) was 28.1 kg/m2. Neurological exam revealed strength of 5/5 in the bilateral upper and lower extremities, normal sensations and brisk reflexes. Cranial nerves were intact except for ptosis in the right eye but extra ocular movements were intact, her gait was normal and she had no ataxia. Remaining physical examination was within normal limits.

Initial laboratory investigations revealed normal complete blood count, comprehensive metabolic panel, thyroid stimulating hormone (TSH), and cardiac markers. Urine toxicology was negative for common substances of abuse. Electrocardiogram was unremarkable.

**Figure 1:**
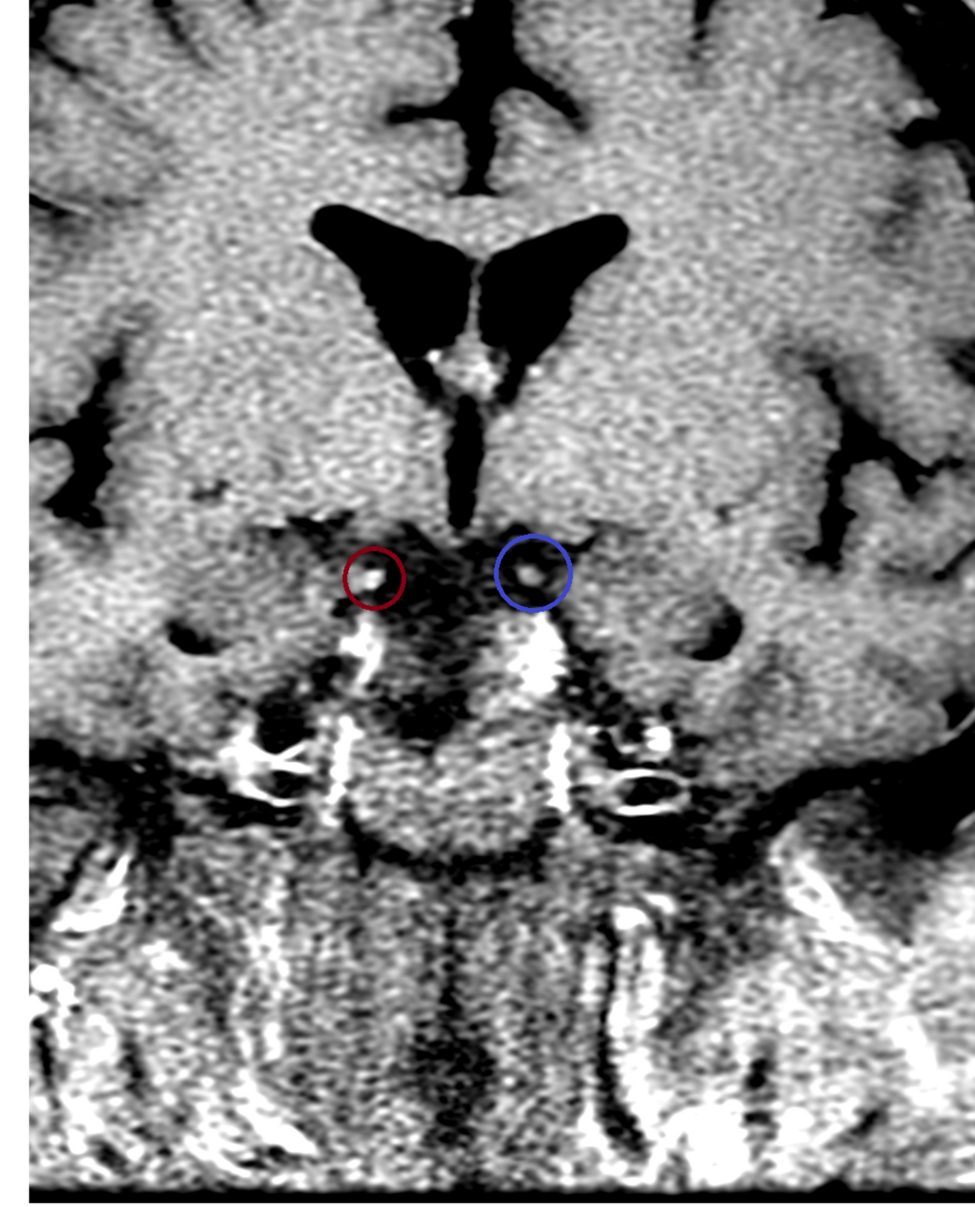
MRI Head T1, Coronal Section. In the figure the red circle shows abnormal enhancement of right CN 3 and blue circle shows normal left CN 3 CN: cranial nerve

**Figure 2:**
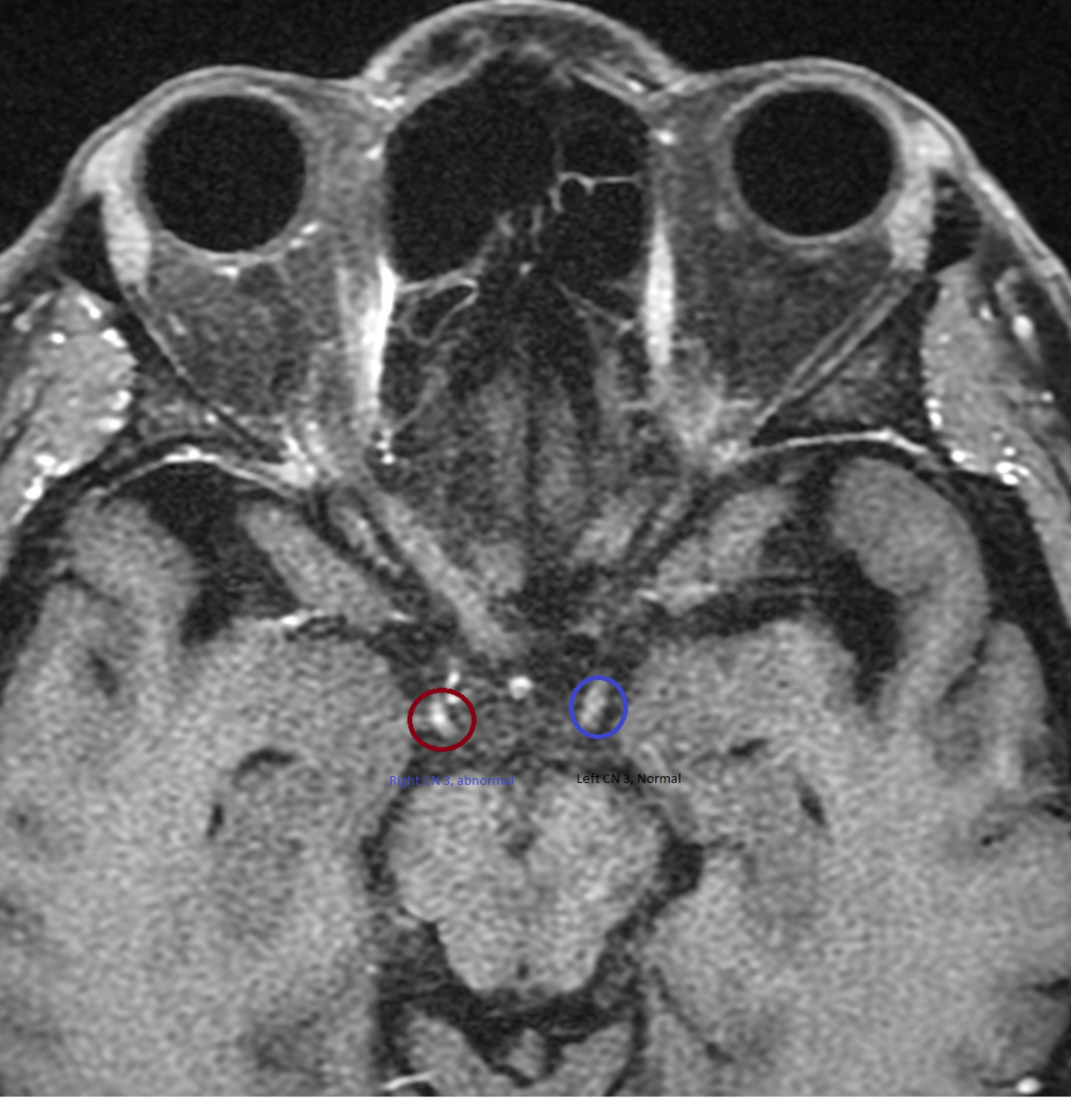
MRI Head T1, Axial Section. In the figure the red circle shows abnormal enhancement of right CN 3 and blue circle shows normal left CN 3 CN: cranial nerve

In the ER, a computed tomography (CT) of the brain showed no acute intracranial pathology and mild mucosal thickening and secretions of the left paranasal sinuses suggestive of acute sinusitis. Neurology was then consulted and recommended additional imaging studies and requested acetylcholine receptor antibodies. Based on initial impression, she was hospitalized for further work up of suspected myasthenia gravis (MG), cerebral vascular accident or cavernous sinus thrombosis. Initial Magnetic Resonance Angiography (MRA) without contrast of the head showed no evidence of infarction with minimal abnormalities. Magnetic Resonance Imaging (MRI) of the head without contrast also showed no evidence of infarction, few nonspecific subcortical cerebral hemispheric white matter lesions suggestive of migraine disease. Left internal carotid cavernous segment cases asymmetric elevation of the left side of the optic chiasm and enhancement of right cranial nerve 3 (Figure 1, 2). A lumbar puncture (LP) was conducted and serology for various autoantibodies including, ganglioside GQ1b antibodies (IgG), acetylcholine receptor antibodies, and muscle-specific tyrosine kinase (MuSK) antibodies was collected without any complications and sent for analysis. Additionally, a trial of 30mg pyridostigmine was given every six hours on admission day two for suspected MG.

Over the next 48 hours, the patient endorsed worsening of the right eye ptosis. Later, she developed an ataxic gait and ophthalmoplegia. GBS was considered and cerebrospinal fluid (CSF) testing revealed non-significant findings (Table 1). Negative inspiratory force and vital capacity were normal. On admission day four, pyridostigmine was discontinued and the patient was started on a course of intravenous immunoglobulin (IVIG) 34 gms/day for seven days. CSF serology returned, showing acetylcholine receptor antibody levels were <0.30 and anti-MuSK levels were unremarkable, which confirmed that the patient did not have myasthenia gravis (Table 2). Anti-GQ1b antibodies were reported positive with a titer value of 1:3200, which confirmed the diagnosis of MFS. Patient was also noted with sensory defect on nerve conduction studies. By day three of IVIG, the patient endorsed significant symptomatic improvement. After completing a course of IVIG for seven days, the patient was discharged on admission day 10 with significant resolution of symptoms. She was later seen in clinic one week after being discharged and had minimal residual ptosis. The patient overlooked numerous follow-ups but was constantly contacted over the phone by hospital staff. The patient reported resolution of her symptoms.

**Table 1:** Cerebrospinal Fluid (CSF) Analysis PCR: polymerase chain reaction, VDRL: Venereal Disease Research Laboratory test

Cerebrospinal Fluid (CSF)	Results	Reference Range
Appearance	Clear	—
white blood cells (WBC)	0	0-5
red blood cells (RBC)	1	0
Bacterial Antigen	No organisms present	—
Gram Stain	No organisms present	—
Glucose	78 mg/dL	40-80 mg/dL
Protein, total	30 mg/dL	15-60 mg/dL
Cryptococcal Antigen	Not detected	—
VDRL	Non-reactive	—
Viral Culture	No viruses isolated	—
Lactic Acid Dehydrogenase	17 units/L	<40 units/L
Cytomegalovirus Ab DNA-PCR	<200 IU/mL	<200 IU/mL
Fungal Culture	No Fungi isolated in 28 days	—

**Table 2:** Antibody Titers IgG: immunoglobulin G

Antibodies	Results	Reference Range
Acetylcholine Receptor Antibody	<0.30 nmol/L	≤0.30 nmol/L
Muscle-Specific Tyrosine Kinase Antibody	<1.0 U/mL	<1.0 U/mL
Ganglioside GQ1B IgG Antibody	1:3200	< 1:100

## Discussion

Miller Fisher syndrome clinically presents as ophthalmoplegia, ataxia, and areflexia. It is commonly thought of as a rare variant of Guillain Barre syndrome, an ascending demyelinating disease. The disease is typically found to occur after a viral illness, more commonly after a URI [2,6]. Viral prodromes have been reported in 71.8% of patients who develop MFS [6]. Other additional symptoms that have been found to occur are paresthesia, dysesthesia, bladder dysfunction, and weakness.

The incidence of Miller Fisher depends on the location of the patient, however in the Western Hemisphere, is about 1-5% of GBS [2-4]. It is more commonly found in males than females with a ratio of 2:1 with an average age of onset of 43 years of age [6]. Miller Fisher is a clinical diagnosis confirmed by the presence of anti-GQ1b antibodies [7]. The specificity of these antibodies has been shown to be 95% [8].

The patient being reported, a 44-year-old female, first presented with right sided unilateral ptosis. Further investigation found that the patient previously experienced diplopia and blurry vision which resolved in two days. Diplopia is a common initial symptom of MFS with an incidence of 38.6% [6]. However, it is an uncommon finding in literature that the diplopia will self-resolve within two days of onset. The initial onset of symptoms is more commonly seen as a progressive diplopia than the symptoms seen in the current case. Additionally, unilateral ptosis is another uncommon finding in literature. A patient presenting with isolated unilateral ptosis with positive anti-GQ1b antibodies has not been easily identified in literature. Jindal et al. reported a case of isolated bilateral ptosis without ataxia, however unlike the present case, areflexia was found [9]. Ptosis is a common finding accompanying the classic triad of symptoms in MFS with 47-58% of patients experiencing ptosis [4,6].

Testing for anti-GQ1b antibodies demonstrated a positive titer value of 1:3200, confirming the diagnosis of Miller Fisher Syndrome. The pathophysiology of ophthalmoplegia may be explained by the high concentrations of anti-GQ1b antibodies found in cranial nerves 3, 4, and 6 [3]. These antibodies, when present, have been found to produce a neuromuscular block when associated with MFS, which may indicate the etiology of the clinical symptoms [10]. While not initially present, ataxia developed later in the clinical course of our patient. Like ophthalmoplegia, antiGQ1b antibodies are also implicated in the development of ataxia. Ataxia in this condition has been described as either due to a sensory deficit or due to a central cause in the cerebellum. Anti-GQ1b antibodies have been found in high concentrations in the IA afferents of muscle spindles [11]. If the muscle spindles are affected, there may be an inability to sense proprioception leading to ataxia. Cerebellar causes of ataxia are restricted to one study. This study found selective staining in the cerebellum by sera in patients with MFS that had positive anti-GQ1b titers [12].

MRI can also be an important tool for the exclusion of other diseases and the diagnosis of MFS. Neuroimaging is typically normal and in one study, 99% of the participants were found to have normal imaging for patients with MFS [4,11]. While normal, imaging can still be an important tool to exclude other potentially fatal diseases. In our patient, T1 coronal and axial MRI Brain demonstrated increased uptake on the right cranial nerve 3 (Figure 1 and Figure 2). This could be due the high concentrations of anti-GQ1b antibodies in this cranial nerve.

The treatment of MFS is similar to that of GBS. Treatment modalities include corticosteroids, plasmapheresis, and intravenous immunoglobulin (IVIG). Initial treatment commonly includes the use of plasmapheresis two to six times every other day, 500mg of corticosteroids for five days, and an IVIG regimen of 0.4g/kg for five days [13-15]. The efficacy of these drugs has not been thoroughly studied to date, however intravenous immunoglobulin and plasmapheresis have been shown to have no effect on the overall outcome of the disease [4,16]. This may presumably be due to the high rate of spontaneous recovery of patients with MFS [4,16]. Treatment with corticosteroids is not recommended due to no perceived benefits and the possibility of delaying recovery [15]. Resolution of symptoms with or without treatment typically takes between two weeks and three months with a complete resolution after six months after the initial onset of symptoms occurs [2,3]. 

Most patients show a complete resolution of all symptoms [6]. However, some symptoms may persist in a few cases. Of these symptoms, areflexia is the most common symptom that persists followed by ataxia and ophthalmoplegia. Less common symptoms that have been found to persist are facial weakness, psychic changes, and tremor [6]. Very rarely, it has been reported that MFS may progress to an advanced portrayal of the disease that mimics GBS. Patients may experience more serious complications such as coma, ballistic movement, dysautonomia cardiomyopathy, and the need for mechanical ventilation [4].

## Conclusions

The current discussion demonstrates a case of self-resolved diplopia with subsequent isolated unilateral ptosis. Miller Fisher Syndrome (MFS) is an uncommon form of GBS that is typically seen after upper respiratory viral illnesses. The presence of ophthalmoplegia presenting as ptosis, diplopia, and blurry vision can be an initial presentation of MFS. A thorough history is vital in establishing a differential diagnosis of other possible causes of demyelinating disorders. Investigative tools of diagnosis include the use of anti-GQ1b antibodies and neuroimaging. These methods can help providers rule out other causes and confirm the diagnosis of MFS. Providers should have a high index of suspicion for Miller Fisher Syndrome in their differential of ophthalmoplegia, especially if presented along with ataxia and areflexia.
